# Scavenger Receptor SREC-I Mediated Entry of TLR4 into Lipid Microdomains and Triggered Inflammatory Cytokine Release in RAW 264.7 Cells upon LPS Activation

**DOI:** 10.1371/journal.pone.0122529

**Published:** 2015-04-02

**Authors:** Ayesha Murshid, Jianlin Gong, Thomas Prince, Thiago J. Borges, Stuart K. Calderwood

**Affiliations:** 1 Molecular and Cellular Radiation Oncology, Beth Israel Deaconess Medical Center, Harvard Medical School, Center for Life Sciences, 3 Blackfan Circle, Boston, Massachusetts, United States of America; 2 Stress Response Center, Boston University Medical Center, Boston, Massachusetts, United States of America; 3 School of Biosciences and Biomedical Research Institute, Pontifícia Universidade Católica do Rio Grande do Sul (PUCRS), Porto Alegre, RS, Brazil; Purdue University, UNITED STATES

## Abstract

Scavenger receptor associated with endothelial cells I (SREC-I) was shown to be expressed in immune cells and to play a role in the endocytosis of peptides and antigen presentation. As our previous studies indicated that SREC-I required intact Toll-like receptor 4 (TLR4) expression for its functions in tumor immunity, we examined potential interactions between these two receptors. We have shown here that SREC-I became associated with TLR4 on binding bacterial lipopolysaccharides (LPS) in RAW 264.7 and HEK 293 cells overexpressing these two receptors. The receptors then became internalized together in intracellular endosomes. SREC-I promoted TLR4-induced signal transduction through the NF-kB and MAP kinase pathways, leading to enhanced inflammatory cytokine release. Activation of inflammatory signaling through SREC-I/TLR4 complexes appeared to involve recruitment of the receptors into detergent-insoluble, cholesterol-rich lipid microdomains that contained the small GTPase Cdc42 and the non-receptor tyrosine kinase c-src. Under conditions of SREC-I activation by LPS, TLR4 activity required Cdc42 as well as cholesterol and actin polymerization for signaling through NF-kB and MAP kinase pathways in RAW 264.7 cells. SREC-I appeared to respond differently to another ligand, the molecular chaperone Hsp90 that, while triggering SREC-I-TLR4 binding caused only faint activation of the NF-kB pathway. Our experiments therefore indicated that SREC-I could bind LPS and might be involved in innate inflammatory immune responses to extracellular danger signals in RAW 264.7 cells or bone marrow-derived macrophages.

## Introduction

SREC-I (scavenger receptor associated with endothelial cells) is the product of the *SCARF1* gene and is a member of the class F family of scavenger receptors (SR), transmembrane proteins with roles in endothelial cell biology and the immune response [1–4]. Like other SR, SREC-I was shown to bind a spectrum of ligands, including the modified proteins acetylated low density lipoprotein and products such as fungal pathogens [5, 6]. SREC-I also bound heat shock protein 90 (Hsp90)-antigen/peptide complexes and thus transmitted the immunostimulatory effects of these chaperone-antigen complexes into antigen presenting cells [2]. Our previous studies also indicated roles for Toll Like Receptors (TLRs) and an associated adaptor molecule MyD88 (myeloid differentiation primary response 88 protein) in the immune effects of HSP vaccines [3].

TLR4 was shown previously to induce inflammatory signaling when bound to LPS derived from Gram negative bacteria [7]. Sequence analysis showed that TLR4 contains an intracellular TIR domain (Toll/IL-1 receptor (TIR) homology domain) shared with the IL-1R, a motif involved in signal transduction [7]. All TLRs were shown to belong to the PRR (pattern recognition receptor) class, shown to recognize pathogen-associated molecular patterns (PAMPs) and thus contribute to innate immunity [8, 9]. Each member of the TLR family has been shown to be distinct in recognizing unique PAMPs derived from different organisms and selectively launching inflammatory signals [7, 8]. After exposure to LPS, TLR4 was shown to stimulate inflammatory gene expression by activating transcription factors including NF-kB, IRF3, NF-IL6 and AP-1 [10, 11]. Such transcriptional activation led to, in turn, the expression and secretion of cytokines, chemokines, type I interferons (IFN-1) and other proinflammatory mediators. However, TLR4 did not bind directly to LPS and was instead shown to rely on primary cell surface receptors, most notably CD14 to associate with the ligand [12, 13]. In addition, the protein MD2 was associated with TLR4 on the cell surface and conferred responsiveness to LPS [14]. However, CD14 did not appear to play an exclusive role in LPS responses and a fraction of the TLR4 activity was observed even under CD14 knockout conditions [15]. Recent studies suggested that SR could interact with TLR4 and mediate inflammatory signaling under some conditions [16]. We have focused on SREC-I in this regard, as our studies have shown this receptor to be involved in antitumor immunity in functional association with TLR2 and TLR4 [3, 17].

In the present study, we asked if SREC-I could interact directly with TLR4 to modify inflammatory signaling and cytokine expression. We showed that exposure to either LPS or the SREC-I ligand Hsp90 initiated profound levels of association of SREC-I with TLR4. In addition, SREC-I was able to mediate LPS-induced TLR4 signaling even in the absence of CD14, suggesting that this SR could act as a receptor for LPS. Although LPS and Hsp90 both triggered SREC-I-TLR4 interactions, LPS was more efficient in stimulating inflammatory signaling. Interestingly, ligand bound SREC-I appeared to play a dominant role in the intracellular localization of TLR4. Activation of SREC-I led to the sequestration of TLR4 in lipid microdomains enriched in cholesterol and signaling molecules such as c-src and Cdc42. Through this pathway, SREC-I appeared to mediate a component of LPS-induced cytokine release in macrophages.

## Methods and Materials

Experiments, where possible employed cells maintained in tissue culture. Some experiments however required fresh primary macrophages for physiological relevance. There were no similar methods or models available for these experiments and the methods and models used are the ones that are more effective for this immunological based treatment strategy. Approaches to animal experimentation were based on guidelines taken from the Weatherall Report- “The use of non-human primates in research.” Experiments are also conducted during the week so that lab personnel and ARF staff could adequately monitor mice. The animals were sacrificed humanely and then bones were taken to prepare bone marrow derived macrophages. We did not see any signs of pain and distress in this procedure. The databases of Pubmed, Medline and OVID were searched to determine if there were alternative methodsormodelsfor bone marrow studies for cytokine assayand we were able to find no alternatives. Experiments were approved by the BIDMC Animal Care Use Committee under IACUCC0792012, approved in 2012 and renewed on Nov 6, 2014 as: “The role of HSF1 and Hsp70 on innate immunity.”

### Mice

C57BL/6, wild type (WT) mice were from the Jackson Laboratories, Maine. C57BL/6 TLR4 KO (*tlr4* ^*-/-*^) mice were obtained from S. Levitz (Boston Medical School). Mice were maintained in micro-isolator cages under specific pathogen free condition. C57BL/6 SCARF KO (*scarf* ^*-/-*^) tibia and fibula were a generous gift from Dr. Terry Means (Massachusetts General Hospital).

### Antibodies and Reagents

LPS (*E*. *coli* 0127:B8) was purchased from Sigma-Aldrich (St. Louis, MO). Ultrapure *E*. *coli* K12 (LPS-EK-ultrapure) was purchased from Invivogen, San Diego, CA. Mouse anti-human SREC-I monoclonal antibody was a gift from Dr. H. Adachi (Laboratory of Cellular Biochemistry, Riken, Saitama, Japan) and rabbit monoclonal mouse anti-SREC-I ab was custom synthesized by GenScript (Piscataway, NJ) against specific peptide sequence (TQGTQGSTLDPAGQC). Rabbit polyclonal anti p38, phospho p38, phospho-NF-kB, NF-kB, phospho ERK2/1, ERK2/1, phospho JNK, JNK antibodies were purchased from Cell Signaling Inc. The inhibitors and chemicals Cytochalasin D, methyl beta cyclo dextrin (MβCD), 4-amino-5-(4-methylphenyl) -7-(t-butyl) were from Sigma-Aldrich and pyrazolo [3,4-d]-pyrimidine (PP1) was from EMD Millipore Corp., Billerica, MA. *Clostridium difficile* toxin B (CTX-B) was purchased from Calbiochem Billerica, MA. Mouse monoclonal anti-FLAG antibody (M2) was purchased from Sigma-Aldrich, St. Louis, MO. FITC labeled anti-CD56 antibody was purchased from BioLegend, San Diego, CA.The ELISA kits were from R&D Systems, Minneapolis, MN and BD Biosciences, San Jose, CA. Alexa labeled LPS was from Life Technologies, Grand Island, NY. Mouse monoclonal anti HA antibody was from Covance, Dedham, MA. Mouse monoclonal anti-TLR4 antibody was from Abcam, Cambridge, MA. Antibodies for macrophage, anti-MAC1 and anti-F4/80 were from eBioscience, San Diego, CA**.** Hsp90 was purified by us from Sf9 cells as described and carefully tested for endotoxin contamination as also described previously [2, 18]. Endotoxin contaminated preparations were discarded. MyD88 and TRIF blocking peptides were purchased from Imgenex Corp., San Diego, CA and InvivoGen, San Diego, CA, respectively. Alexa labeled LPS (*E*. *coli* 0127:B8) was from Sigma-Aldrich, St. Louis, MO. OxLDL was from Biomedical Technologies Inc., Ward Hill, MA. The mouse anti SR-A antibody (2F8) was from Hycult Biotech., Plymouth Meeting, PA. Anti CD14 ab was from Abcam, Cambridge, MA and CD14 neutralizing ab was purchased from R&D Systems, Minneapolis, MN. Anti IRF3 and phospho-IRF3 were purchased from Cell Signaling, Danvers, MA.

### Cells and culture conditions

Wild type HeLa and HEK 293, Raw 264.7 cells were maintained in DMEM (with 4.5 g/L glucose) supplemented with 10% heat inactivated FBS, streptomycin and penicillin. HEK 293 expressing TLR4-CD14-MD2 cells were maintained in the medium described for HEK 293 and HeLa cells with 100 μg/ml Normocin. CHO-SREC-I cells were maintained in F12K media supplemented with 10% heat inactivated FBS, streptomycin and penicillin and 400 μg/ml G418. All cells were maintained in a 5% CO_2_ humidified incubator.

### Bone marrow-derived macrophage preparation

Macrophages were obtained from mouse bone marrow culture using the method described by Weischenfeldt and Porse (2008). Briefly, bone marrow macrophages were enriched by lysis of red cells. Cells were then passed through a cell strainer then grown in L929 conditioned medium for proliferation and differentiation into a homogenous population of mature bone marrow-derived macrophages.

### Plasmids and Transfection

The pcDNA3.1-SREC-I (human) was a generous gift from Dr. H. Adachi. The FLAG-SREC-I construct was constructed from the 3xFLAG-CMV vector. Human and mouse siRNA SREC-I and TLR4 was purchased from Santa Cruz Biotechnology Inc., Dallas, TX.

### SEAP reporter assay

The secreted form of embryonic alkaline phosphatase (SEAP)–NF-kB promoter-reporter assay kit was utilized as a convenient and sensitive method to determine promoter activity in cells transfected with the SEAP expression plasmid. HeLa cells were transfected with plasmids encoding FLAG-SREC-I, TLR4 and the reporter constructs NF-kB-SEAP and CMV- (control expression vector). NF-kB activity was measured indirectly by catalytic hydrolysis reaction of *p*-nitrophenyl phosphate producing a yellow end product that was read spectrophotometrically at 405 nm.

### Western Blotting and Immunoprecipitation

HEK 293 cells expressing TLR4 and SREC-I were treated with or without LPS and Hsp90. Cells were then washed with ice-cold Dulbecco’s phosphate-buffered saline (PBS) and lysed in NP-40 lysis buffer (containing 1% Nonidet P-40, 150 mM NaCl, 1mM EDTA, 1 mM PMSF, 1x HALT protease and phosphatase inhibitor cocktail (Thermo Scientific). For Western blotting, 15–30 μg of protein were resolved by 4–15% gradient SDS-PAGE and transferred to PVDF (polyvinylidenefluoride) membranes. Membranes were immunoblotted with primary antibodies and later secondary antibodies that are HRP-conjugated. The membrane reactions were visualized by Perkin Elmer enhanced chemiluminescence reagents. For immunoprecipitation, 1 mg of cell extract was incubated with 5 μg of the selected antibody for 2 hours at 4°C followed by incubation with 20 ul of protein A (50% slurry, GE healthcare) plus-sepharose beads for either 2 hours at room temperature or overnight at 4°C. The beads were then washed with NP-40 lysis buffer and complexes were eluted by boiling in Laemmle sample buffer.

### Isolation of Lipid microdomain

Raw 264.7 cells (80–90% confluent) were incubated with or without ice cold LPS (1 μg/ml) for 3–5 minutes and then lysed with lysis buffer (Sigma) containing, 1% Triton X-100 and protease inhibitor cocktail (Sigma). Immediately before the assay, 1 ml of lysis buffer containing 1% Triton X-100 for each gradient was prepared on ice. The density gradient was made of 4 layers of OptiPrep with different concentrations: 35%, 25%, 20% and 0%. Lower layer (35% OptiPrep) contains the cell lysate. The 35% Gradient layer mixed with cell lysate was placed at the bottom of pre-cooled ultracentrifuge tube and then the centrifugation performed at ~200,000xg using TFT 65.13 rotor for 4 hours at 4°C. Fractions including the fraction containing lipid microdomain were gently removed.

*ELISA*: IL-6, TNF-α were purchased from R&D Biosystem, Minneapolis, MN, BD Biosciences, San Jose, CA, PBL interferon sources, IFN-β was from West Logan, Utah. ELISA of cell media for cytokine release was performed for each cytokine according to manufacturer’s protocol using appropriate antibody.

### LPS binding experiments by flow cytometry

Cells were preincubated without or with mBSA (50 mg/ml) or Alexa labeled LPS (1 μg/ml) at 4°C in FACS buffer (PBS containing 0.1% BSA and 0.05% NaN3). The cells were then washed with FACS buffer twice, fixed with 4% paraformaldehydefor 10 min, and analyzed for binding of Alexa-LPS with FACSCanto II and FACSDiva (BD Biosciences, San Jose, CA).

### Immunofluorescence and Microscopy

Cells were labeled or incubated with Alexa-LPS or FITC-anti-CD59 antibody on ice or at 37°C for 20–30 minutes then fixed with 4% para-formaldehyde and either permeabilized using 0.1% Triton X 100 (for visualizing intracellular proteins) or not (for surface expression or binding) using 0.1% Triton X 100. Cells were stained with primary antibodies and then washed three times with 1x PBS and stained again with fluorophore-conjugated secondary antibodies. Fluorophores were visualized using the following filter sets: 488 nm excitation and band pass 505–530 emission filter for Alexa 488; 543 nm excitation and band pass 560–615 for Cy3/Alexa 564; and 633 excitation and long pass 650 for Cy5.

## Results

### SREC-I was associated with TLR4 in the presence of LPS and was present in LPS-TLR4 complexes

**Fig 1:**
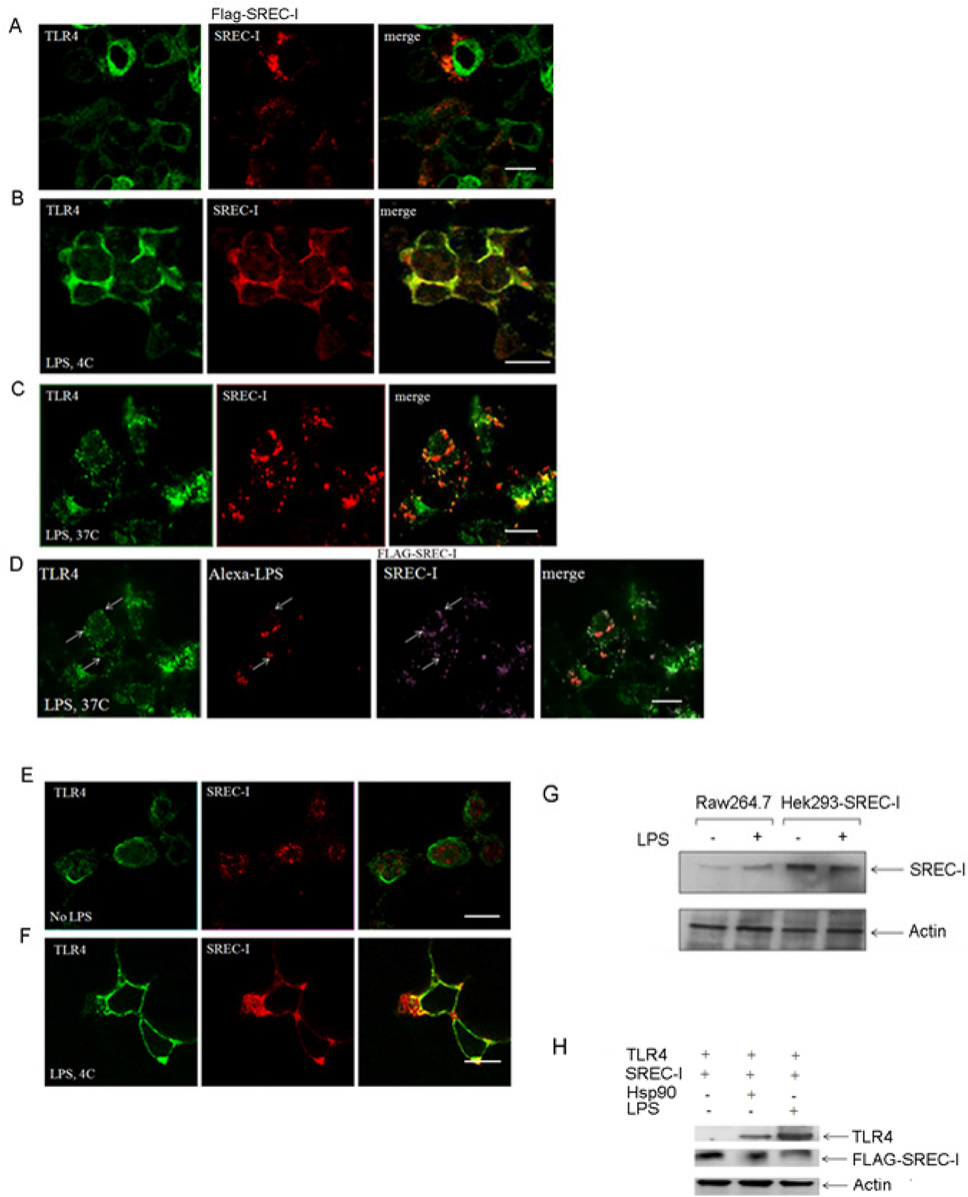
Ligand-bound SREC-I colocalized with TLR4 after LPS treatment. **A,** SREC-I and TLR4 did not interact in the absence of LPS. Raw 264.7 cells were transfected with FLAG-SREC-I for 22 hours. Cells were then fixed and stained with anti TLR4 ab (green) and anti-FLAG ab (red). **B,** TLR4 colocalized with SREC-I in the presence of LPS. Raw 264.7 cells overexpressing FLAG-SREC-I were exposed to LPS (1 μg/ml) for 20–30 min at 4°C. Cells were then fixed and stained for TLR4 (green) and FLAG (red). Percent colocalization with or without LPS is shown in the adjacent histogram. **C, D,** LPS, TLR4 and SREC-I were internalized at 37°C. Raw 264.7 cells overexpressing FLAG-SREC-I were incubated with Alexa LPS (1 μg/ml) at 4°C for 20 mins and then medium was replaced with warm medium. Cells were then incubated at 37°C for 10–15 mins. Cells were fixed and stained for TLR4 (green, C, D) and FLAG-SREC-I (red in C, purple in D). Alexa LPS was shown in red (D). **E, F**, Endogenous TLR4 and SREC-I did not colocalize in the absence of LPS. Raw 264.7 cells were treated for 30 mins with LPS (1 μg/ml) and then fixed and stained for TLR4 (green) and SREC-I (red). **E,** TLR4 and SREC-I colocalize in the presence of LPS. Cells were labeled with LPS (1μg/ml) for 20–30 minutes at 4°C then fixed and stained for TLR4 (green) and SREC-I (red) (F). **G,** SREC-I expression level in Raw 264.7 cells and in cells overexpressing FLAG-SREC-I. **H,** SREC-I interacted with TLR4 physically in the presence of LPS (1 μg/ml). HEK 293 cells expressing FLAG-SREC-I and TLR4s were treated with or without Hsp90 or LPS. FLAG-SREC-I was then immunoprecipitated (IP) with FLAG ab. The IP complex was separated with SDS-PAGE and blotted for TLR4. The amount of FLAG-SREC-I immunoprecipitated was determined and β-actin was used as a loading control. All images were representative of 3 different planes from each sample. Each experiment was performed 3 times reproducibly. Scale bar, 5 μm.

We first investigated the effects of LPS on SREC-I the intracellular localization of SREC-I (Fig 1). As SREC-I was shown to be expressed at low levels in resting macrophages (B. Zhou & SK Calderwood, unpublished data), we carried out overexpression of the receptor in the mouse macrophage cell line Raw 264.7 to permit effective visualization by immunofluorescence. We then incubated these cells with *Escherichia coli* derived LPS (1 μg/ml) at 4°C, fixed the cells and analyzed SREC-I and TLR4 localization by confocal microscopy. Prior to LPS exposure, SREC-I was detected largely in the cytosol whereas TLR4 was mostly membrane-associated; minimal overlap between these fluorescence signals was observed (Fig 1A). However, TLR4 and SREC-I became partially coincident on the cell surface in the presence of LPS as indicated by the strong overlap in fluorescence patterns in cells at 4°C (Fig 1A and 1B). We then investigated internalization of TLR4 and SREC-I in LPS treated cells after warming the medium to 37°C (Fig 1C). LPS exposure prompted internalization of both receptors at 37°C and their relocation to intracellular vesicles, with some of these structures marked both by anti TLR4 and anti-FLAG antibodies (for SREC-I) (Fig 1D). We also showed fluorescent, Alexa-labeled LPS to be localized in intracellular vesicles containing TLR4 and SREC-I at 37°C, suggesting partial co-internalization of SREC-I, LPS and TLR4 (Fig 1C and 1D). As TLR4 was not shown previously to bind directly to LPS, these results suggested SREC-I to be a recognizing receptor for LPS and that could induce the recruitment of TLR4 to SREC-I marked regions on the cell surface. Although CD14 is a well-established LPS recognizing molecule cooperating with TLR4, our experiments suggested that SREC-I was also capable of recognizing the endotoxin and interacting with TLR4.

To further confirm receptor co-association in the presence of LPS (1 μg/ml) under conditions of native expression, we next activated Raw 264.7 cells by pre-exposure to LPS (1–5 ng/ml) to increase expression of SREC-I to levels detectable by immunofluorescence (3) before experiment. After recovery from the activating LPS exposure for 24hr, cells were then transiently re-exposed to LPS (1 μg/ml) for 20–30 minutes on ice, then fixed and stained for SREC-I and TLR4 (Fig 1E and 1F). As with the overexpression studies, TLR4 and SREC-I appeared to become co-localized on the plasma membrane in the presence of LPS. We saw minimal evidence of co-localization of the receptors in controls in the absence of the endotoxin. Levels of SREC-I in Raw 264.7 cells used in (Fig 1E and 1F) and in FLAG-SREC-I overexpressed HEK 293 cells used in the experiment were shown in Fig 1G.

To investigate biochemically a physical interaction between TLR4 and SREC-I we next used HEK 293 cells (low in *S*cavenger *R*eceptors, *SR* expression) for co-immunoprecipitation studies. These cells were used rather than Raw 264.7 to avoid the potentially complicating presence of other SR family members on SREC-I interaction with TLR4 (Fig 1H). While we found minimal evidence of co-precipitation of FLAG-SREC-I and TLR4 in control, unstimulated cells (lane 1) these proteins interacted substantially in the presence of either LPS or another SREC-I ligand, Hsp90 (Fig 1H, lanes 2, 3). These experiments indicated binding of SREC-I and TLR4 only in the presence of either TLR4 or SREC-I ligands such as Hsp90.

For these studies, we used LPS at a concentration of 1 μg/ml for the ligand-receptor localizationas this amount of LPS could bring its receptors CD14/SREC-I to the plasma membrane and bind efficiently and could easily be detected when dye-tagged.

### SREC-I enhanced LPS-TLR4 mediated NF-kB activity

**Fig 2:**
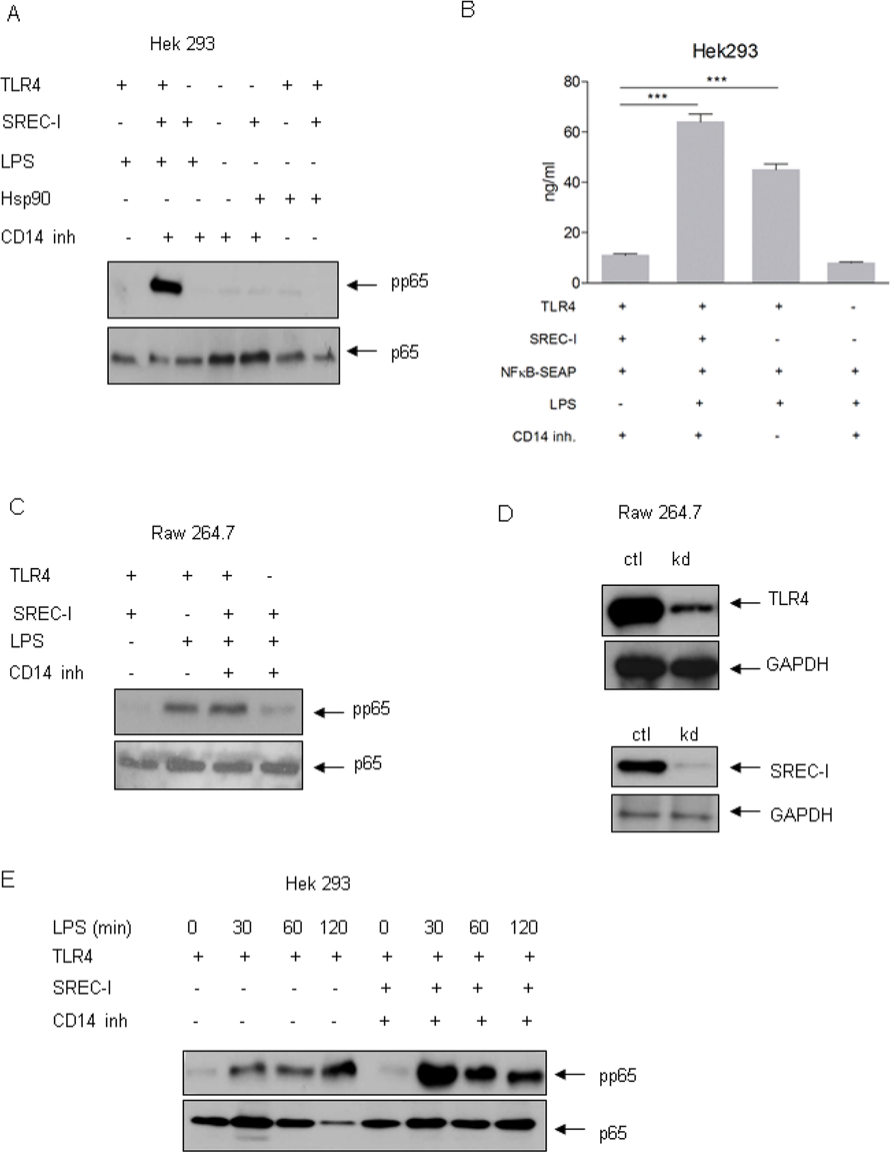
SREC-I supported LPS-TLR4 mediated NF-kB (phospho-p65). **A,** Phospho-p65, (S536/Rel) level was increased in cells expressing SREC-I with TLR4 in the presence of LPS. HEK 293 cells expressing TLR4-MD2-CD14 and/or SREC-I, SREC-I only were treated with or without LPS (1 μg/ml) or Hsp90 for 5–7 hours. Cell lysates were then collected and SDS-PAGE was performed. Phospho-p65 levels were measured. Total level of p65 was measured in the same lysate. Total p65 level was determined. **B,** HEK 293 cells expressing SREC-I and TLR4 or TLR4 only were transfected with NF-kB-SEAP and incubated with LPS (1 μg/ml) for 5 hours. NF-kB activity was measured as instructed by NF-kB-SEAporter assay kit. **C,** Raw 264.7 cells were transfected with siRNA for SREC-I/TLR4 for 72 hours and incubated with LPS (1 μg/ml) with or without CD14 neutralizing peptide (inhibitor). Phospho-p65 level is increased with LPS incubation in cells expressing both TLR4 and SREC-I. **D,** Raw 264.7 cells were transfected with ctl (scr) siRNA or TLR4 siRNA/SREC-I siRNA for 72 hours. Cell lysates were isolated and later SDS-PAGE was performed. **E,** HEK 293 cells expressing TLR4, MD-2, CD14 or TLR4, MD-2, CD14 and SREC-I were incubated with LPS (1 μg/ml) for indicated time. Cells lysates were collected and equal amount of protein was loaded for SDS-PAGE experiment. For blocking CD14 activity, cells were treated with 10 μg/ml of anti CD14 neutralizing antibody. Error bars in graph show S.D. between three replicate experiments. *P* <0.0001 values were generated by ANOVA using the Bonferroni post-test.

As NF-kB is the most potent proinflammatory transcription factor, we examined the potential role of SREC-I in its activation by LPS. TLR4 activation has been shown to trigger activation of NF-kB. We next examined therefore the potential activity of SREC-I in LPS triggered NF-kB signaling in HEK 293 cells. As mentioned above, in contrast to Raw 264.7, HEK 293 cells were shown to be deficient in most SR family members (including SREC-I), thus SREC-I-specific effects could be examined in isolation in transfectants. This HEK 293 cell line stably expressed CD14, TLR4 and MD2; CD14 independent signaling was determined using CD14 blocking peptides (CD14-inh, 10 μg/ml), while the role of TLR4 was probed using RNA interference. The CD14 blocking peptide ab was first tested in THP1 cells by its ability to neutralize LPS-induced TNF-α secretion. The ab neutralized >60% of LPS induced cytokine release in this cell line. NF-kB activation was assayed by determining phosphorylation of its *trans*-activating subunit p65/Rel on serine 536 [19]. We then compared LPS-induced NF-kB signaling in cells expressing TLR4 and the LPS recognizing and signaling adaptor proteins CD14 and MD2 (Fig 2A, lane 1) without (lane 1) or with SREC-I (lane 2). LPS activated NF-kB in the absence of CD14 but with SREC-I expression (lane 2). Minimal signaling was observed in the absence of TLR4 with or without SREC-I (lanes 3, 4). This experiment showed therefore a role for SREC-I as recognizing receptor for LPS that could interact with TLR4. We also asked if exposure to an alternative SREC-I ligand, Hsp90 could trigger p65 phosphorylation (Fig 2A, lanes 5–7). However, minimal increases in phospho-536-p65/Rel levels were induced by Hsp90 even in cells expressing SREC-I plus TLR4 (Fig 2A).

Next, we further examined the role of SREC-I as a recognizing receptor for LPS and a partner for TLR4 using an NF-kB reporter assay. We assayed NF-kB activity in the CD14, TLR4 and MD2 producing HEK 293 cells with or without SREC-I expression (Fig 2B). We again found that exposure to LPS could activate NF-kB additively with SREC-I and TLR4 co-expression (Fig 2B). In the absence of LPS, reporter activity was minimal but was activated by the endotoxin in cells expressing either SREC-I+TLR4 (lane 2) or CD14 +TLR4 (lane 3). Activity was minimal in the absence of TLR4 (Fig 2B, lane 4). LPS could also activate NF-kB signaling in the CD14 expressing Raw 264.7 cell line (lane 2) (Fig 2C). In addition, in the presence of CD14 neutralizing antibodies, LPS activated NF-kB when SREC-I was expressed in these cells (Fig 2C, lane 3). However depletion of TLR4 by siRNA inhibited LPS-mediated p65/Rel-serine 536 phosphorylation (lane 4). The expression levels of TLR4 and SREC-I in cells transfected with control (ctl) and sequence specific siRNA (kd) is shown in Fig 2D. We also measured the kinetics of LPS-induced NF-kB activation by probing levels of phospho-536-p65/Rel (pp65) as in A (Fig 2E). In cells expressing TLR4 and CD14, we observed the prolonged activation of p65/Rel-S536 phosphorylation that was exceeded in cells expressing both TLR4 and SREC-I. This increase in p65 phosphorylation in SREC-I expressing cells did not require the CD14 recognition of LPS.

### LPS in SREC-I and TLR4 expressing cells increased MAPK activity

**Fig 3:**
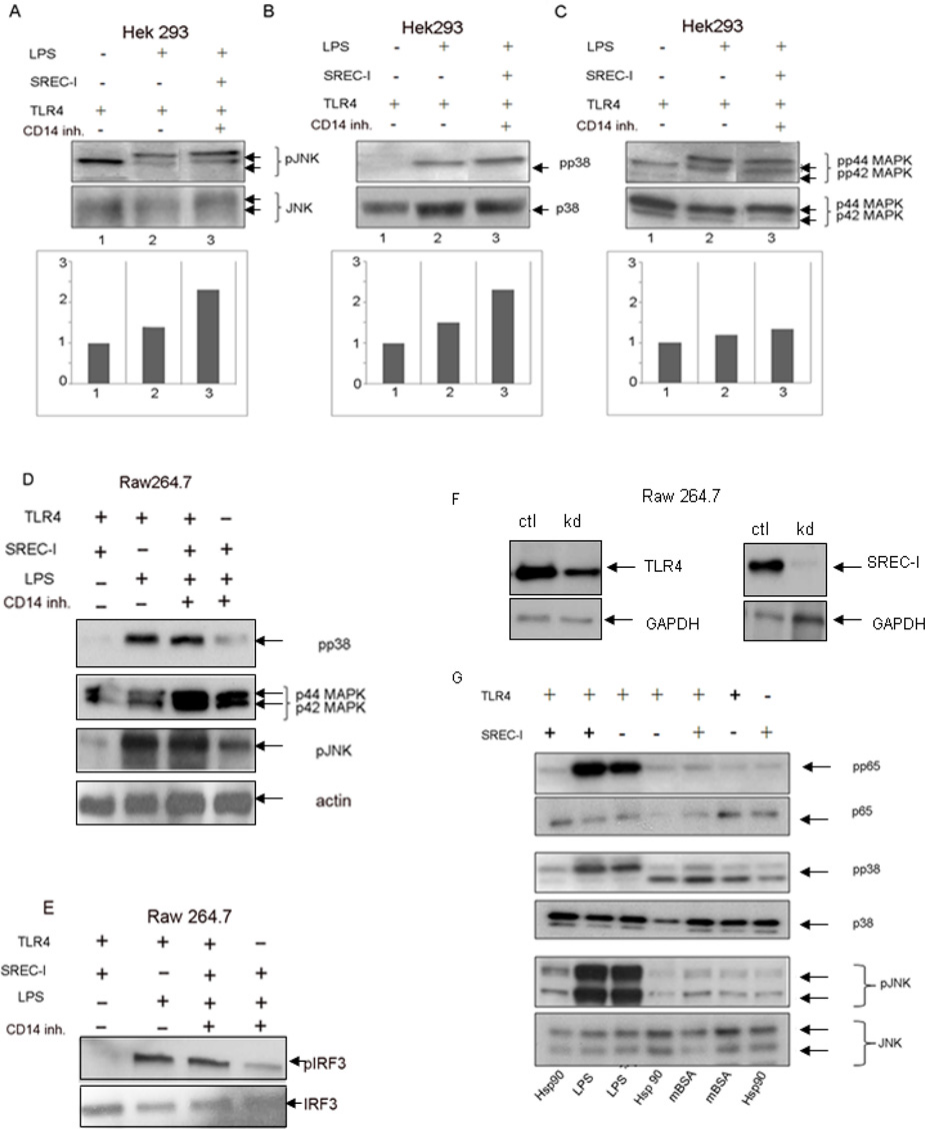
SREC-I expression led to enhanced NF-kB and MAPK activities in cells expressing TLR4 in the presence of LPS. **A, B, C,** SREC-I could increase LPS-TLR4 activation of MAPK. HEK 293 cells expressing TLR4-MD2-CD14, TLR4-MD2-CD14-SREC-I were incubated with LPS (1 μg/ml) for 5 hours (CD14 neutralizing peptide added to SREC-I incubation). Cell lysates were collected and levels of phosphorylated JNK (A), p38 (B), ERK1/2 MAPK assayed (C). **D,** SREC-I was shown to activate LPS-TLR4 induced MAPK signaling. Raw 264.7 cells were transfected with siRNA SREC-I/TLR4 and then incubated with or without LPS in the presence of CD14 neutralizing ab (inhibitor) or not. Cell lysates were collected and subjected to SDS-PAGE and Western Blotting. **E,** SREC-I was involved and IRF3 activity in the absence of CD14. Raw 264.7 cells were treated as in D and then cell lysates were collected and subjected to SDS-PAGE and Western blotting with appropriate antibodies. **F,** Raw 264.7 cells were transfected with ctl siRNA (scr) or TLR4/SREC-I for 72 hours. Cell lysates were collected and equal protein was subjected to SDS-PAGE and Western Blotting. **G,** Phospho-p65 levelswere high in cells expressing TLR4-SREC-I. HEK 293 cells expressing TLR4-MD2, SREC-I-MD2, TLR4-MD2-SREC-I were incubated with mBSA (10 μg/ml), LPS (1 μg/ml), Hsp90 (10 μg/ml), Hsp90 (10 μg/ml) for 3–5 hours. Cell lysates were collected and levels of phospho-p65, p65, phospho-p38, p38, phospho-JNK and JNK measured. Densitometric analysis of gel intensity was performed using Image J software. Experiments were repeated reproducibly three times.

In the next series of experiments, we asked whether SREC-I could also mediate signaling through the mitogen activated protein kinase (MAP kinase) pathways. The MAPK family members are important in activation of alternative factors in inflammatory cytokine transcription such as AP-1 and NF-IL6 [20]. We therefore examined levels of activated phosphorylated c-jun kinase (JNK), p38 MAPK (pp38) and ERK-MAPK (pp42 MAPK and pp44 MAPK) after LPS stimulation (Fig 3, S1 Fig). Indeed, SREC-I expression was permissive for LPS-induced JNK, p38 and ERK pathways in TLR4 expressing HEK 293 cells even when CD14 activity was deterred by CD14 neutralizing ab (CD14 inhibitor) (Fig 3A, 3B and 3C). We also found that SREC-I could mediate LPS-induced MAPK activity in Raw 264.7 cells (Fig 3D). MAPK activation was sustained by either CD14 (lane 2) or SREC-I (Fig 3D, lane 3). These experiments suggested that SREC-I could maintain TLR4 mediated LPS signaling even in the presence of the CD14 neutralizing peptide as with the earlier experiments on NF-kB signaling (Figs 2 and 3).

In further experiments we examined LPS-TLR4 induced IRF3 activity, which occurs only after TLR4 endocytosis (Fig 3E). This signaling pathway is initiated after LPS-TLR4 undergoes endocytosis through adaptors other than MyD88 and leads to signaling through the transcription factor IFN, a molecule that regulates transcription of Interferon-β (IFN-β) [21]. IFN-β plays a key role in antigen presentation and adaptive immunity [22]. We thus aimed to determine if SREC-I was involved in internalization of TLR4 induced signaling activity initiated from the endosomes. Indeed we found LPS-TLR4 to be endocytosed and to activate IRF3 in both CD14 active cells (Fig 3E, lane 2), as well as CD14 inhibited cells expressing SREC-I (Fig 3E, lane 3). These experiments suggested a potential role for SREC-I in internalizing LPS-TLR4 complexes and activating IFN-β production through phosphorylation and activation of IRF3. The downregulated expression of SREC-I and TLR4 in Raw 264.7 cells with siRNA specific for these two receptors is shown in Fig 3F.

Next, the ability of other ligands for SREC-I, including Hsp90 and maleylated BSA (mBSA) to activate NF-kB, p38-MAPK and c-Jun kinase (JNK) was queried (Fig 3G). However, neither mBSA nor Hsp90 significantly activated the NF-kB or the MAPK pathways in cells expressing SREC-I only, TLR4+CD14 or TLR4+SREC-I (plus CD14 neutralizing ab), suggesting LPS-specific signaling events not duplicated by other SREC-I ligands (Fig 3G). By contrast LPS strongly activated each pathway in positive controls (Fig 3G, second and third lane).

### Downstream factors in TLR4-NF-kB Signaling

MyD88 was first characterized as an essential adaptor for all TLRs [23]. This protein was shown to possess a C-terminal TIR domain through which it interacted with TLR family members [24]. In addition, it was shown that macrophages from MyD88 knock-out mice exhibited minimal responses to LPS, indicating an essential role for this molecule in LPS-mediated signaling [25]. MyD88 was required for robust, SREC-I mediated immune responses to tumor antigens [3]. In addition to MyD88, TLR4 has also been shown to activate NF-kB signaling in association with the adaptor molecule, TRIF (TIR-domain-containing adapter-inducing interferon-β) [26–28]. TLR4 is the only member of the TLR family shown to interact with both MyD88 and TRIF [28]. Thus LPS-TLR4 could mediate downstream signaling in both MyD88-dependent and MyD88-independent (TRIF dependent) manners. Therefore, we next asked whether SREC-I could also interact with TRIF in an LPS-dependent manner.

**Fig 4:**
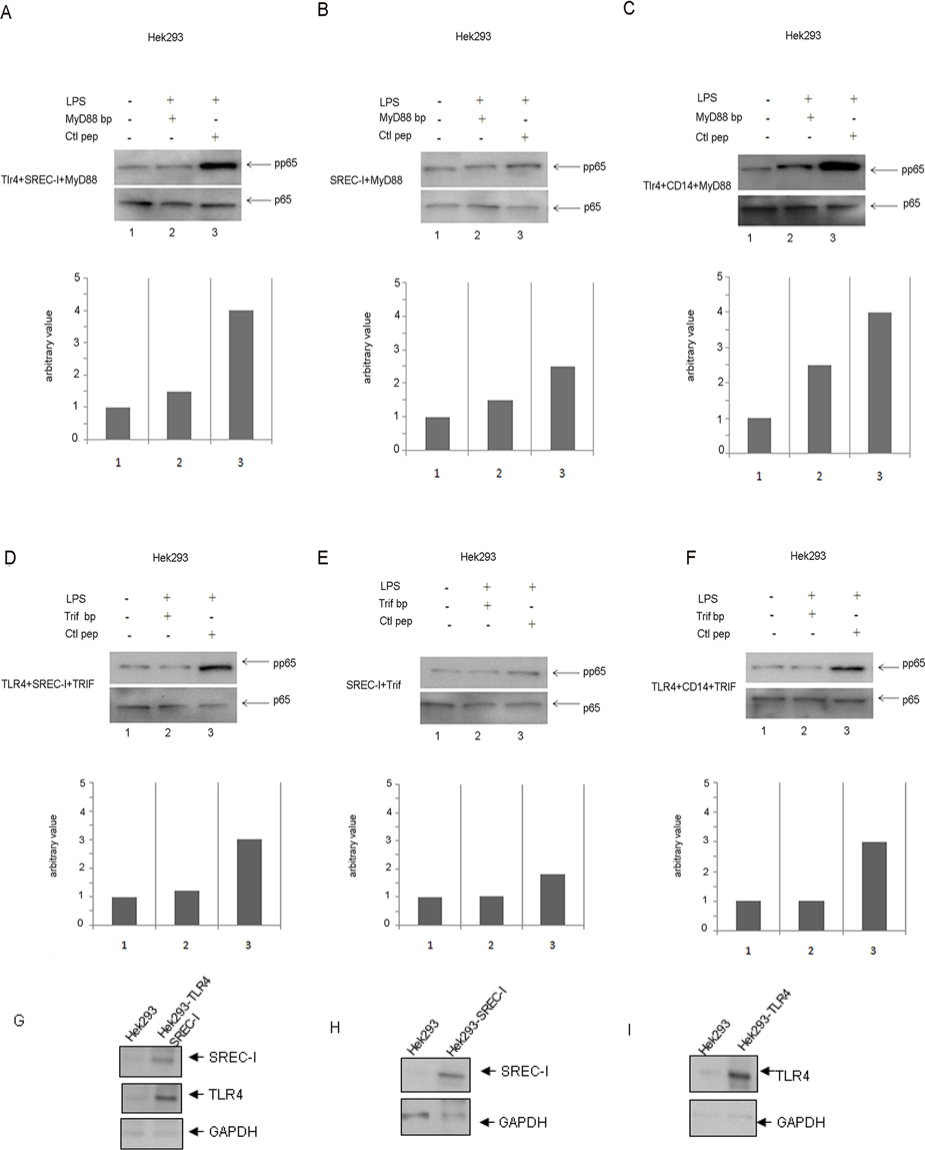
SREC-I requiredadaptor proteins MyD88 and TRIF for downstream NF-kB activation. **A, B, C,** MyD88 was involved in LPS-TLR4-SREC-I (A), LPS-SREC-I (B) or LPS-TLR4 (C) activation of NF-kB. HEK 293 cells expressing TLR4-MD2-SREC-I-MyD88, SREC-I-MD2-MyD88 and TLR4-MD2-MyD88-CD14 were treated with MyD88 blocking peptide (100 μM for 24 hours), or control peptide (100 μM for 24 hrs) as indicated. Cells were then treated with LPS (1 μg/ml) for 5 hours in the presence of blocking peptides. Densitometric analysis of bands in gels is shown. **D, E, F,** TRIF was involved in LPS-TLR4-SREC-I, LPS-TLR4 or LPS-SREC-I activation of NF-kB. HEK 293 cells (+MD2) expressing receptors and adaptor TRIF and CD14 as indicated were incubated with TRIF blocking peptide or control peptide (50 μM for 6 hours) and incubated with LPS for 5 hours. Phospho-p65 levels in cell lysates were then analyzed by SDS-PAGE and Western blotting. TLR4 only cells also express CD14 as shown. Densitometric analysis of band intensity is shown below. Each experiment was repeated 3 times. **G, H, I,** Protein lysates from HEK 293 cells stably expressing TLR4 and SREC-I, TLR4 only and SREC-I only were analyzed by SDS-PAGE and Western blotting. Relative protein expression levels are thus shown in the blot.

We investigated whether the SREC-I-TLR4 interaction led to LPS-mediated NF-kB signaling through either (1) MyD88 or (2) TRIF (Fig 4). We used MyD88 and TRIF blocking peptides to probe the roles of these adaptors. The MyD88 inhibitory peptide used here was shown to block MyD88 signaling by inhibiting its homodimerization whereas the TRIF inhibitory peptide interfered with TLR4-TRIF binding [29, 30]. We compared HEK 293 cells (that normally express MyD88) which were manipulated by transfection or CD14 blocking to produce either: (A) a TLR4 plus SREC-I phenotype, (B) SREC-I alone or (C) a TLR4 plus CD14 phenotype. The densitometric analysis of band intensity from one experiment is shown beneath the immunoblots in Fig 4. In the TLR4 plus SREC-I conditions, LPS activated NF-kB activity (Fig 4A, lane 3) and these effects were inhibited by the MyD88 blocking peptide (Fig 4A, lane 2). Likewise in the presence of CD14 and TLR4, LPS activated NF-kB (Fig 4C, lane 3) and these effects were inhibited by the MyD88 blocking peptide (Fig 4C, lane 2). In cells expressing SREC-I alone, without TLR4 there was less pronounced NF-kB activation that was however also inhibited by the MyD88 blocking peptide (Fig 4B). The control peptide that did not block MyD88 had minimal effects on NF-kB activation (Fig 4A–4C, third lanes). TRIF blocking peptides also inhibited LPS induced activation of NF-kB in a similar way to the MyD88 inhibitory peptide under each condition, except for the cells expressing SREC-I only (Fig 4D, 4E and 4F). These findings suggested that this peptide could inhibit interactions between TLR4 and TRIF (Fig 4D, 4E and 4F). In the experiment under SREC-I alone conditions, activation of NF-kB by LPS was too faint to draw conclusions (Fig 4E).

These results suggested that SREC-I, in addition to CD14 could recognize extracellular LPS and, when bound to this ligand, could further interact with TLR4, and either MyD88 or TRIF to activate NF-kB signaling (Fig 4A–4F). SREC-I alone signaled to NF-kB only very faintly and would likely require TLR4 for significant LPS activation (Fig 4B and 4E). Expression of SREC-I, TLR4 in HEK 293 cells overexpressing TLR4/SREC-I or TLR4 + SREC-I is shown in Fig 4G, 4H and 4I).

### SREC-I was localized to detergent-insoluble lipid microdomains in the presence of LPS

**Fig 5:**
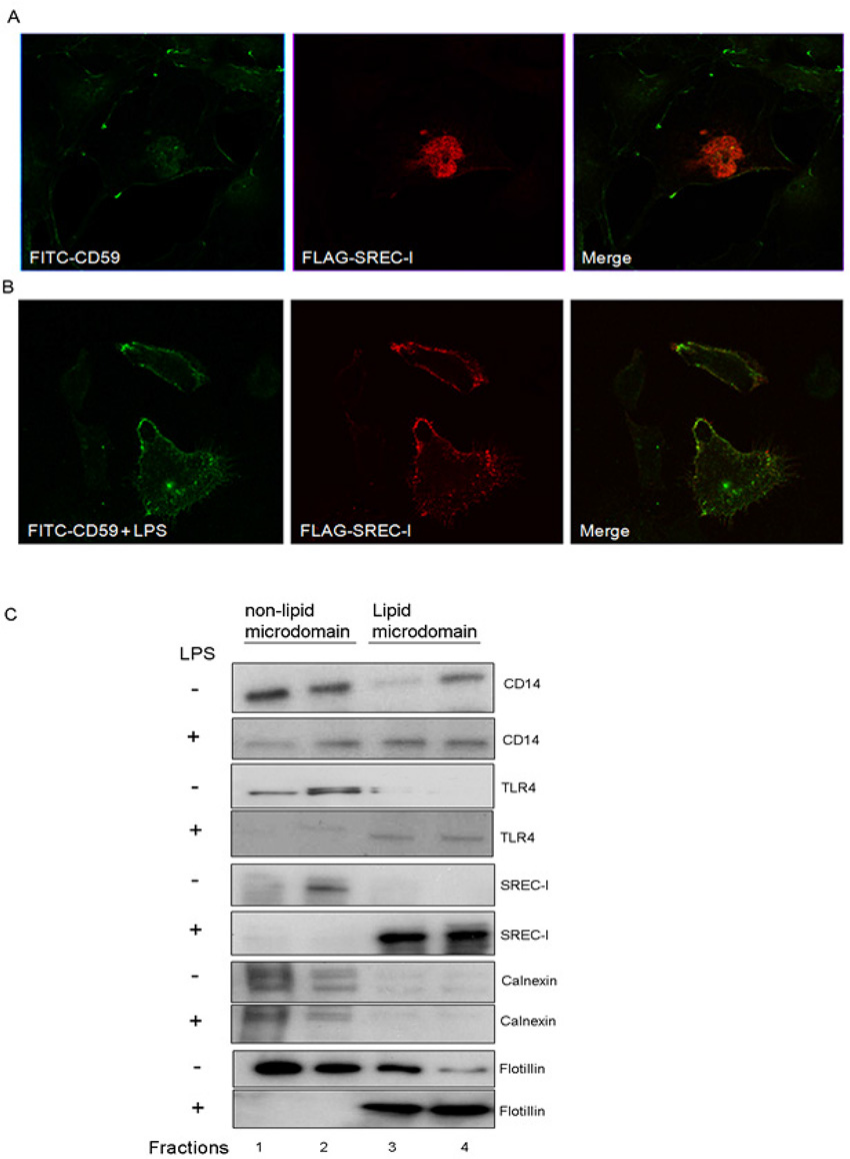
SREC-I became localized in detergent insoluble lipid microdomainsafter LPS exposure. **A,B,** FLAG-SREC-I were localized to lipid microdomains marked by the GPI anchored protein CD59 on the plasma membrane in the presence of LPS (B). HeLa cells expressing FLAG-SREC-I were incubated with LPS (1 μg/ml) and FITC labeled (red, in figure) anti CD59 ab (10 μg/ml for 30 minutes on ice at 4°C). Cells were then fixed on ice and stained for FLAG (green) using anti FLAG M2 ab. FLAG-SREC-I expressing cells were also incubated with FITC-anti-CD59 ab at 4°C for 30 mins. Cells were stained with anti-FLAG ab (secondary antibody, red). Confocal Microscopy was then used to analyze the localization of the proteins. **C,** SREC-I was localized to lipid microdomains after LPS exposure. Raw 264.7 cells were stimulated with LPS (1 μg/ml) or incubated without LPS and lipid microdomains were isolated using *Optiprep* density gradient centrifugation. 1 ml fractions (total 4) were collected from the top of the centrifuge tubes. Fractions containing detergent soluble membrane proteins were also indicated. All fractions were later separated on polyacrylamide gels, electrophoretically blotted and probed with antibodies as indicated. Flotillin was used as marker for lipid microdomain rich fractions (shown in lane 3 and 4).

In previous studies of SREC-I, we showed this receptor to be internalized by a pathway involving segregation of ligand-SREC-I complexes into detergent insoluble lipid microdomains and subsequent endocytosis through the *GPI-anchored protein (GP-AP) enriched early endosomal compartment* (GEEC) pathway [2, 31, 32]. The ligand (Hsp90-peptide complex) uptake and antigen cross presentation were inhibited by antagonists of cholesterol localization, actin polymerization and the small GTPase Cdc42, characteristic of endocytosis through the pathway taken by GPI-AP [2]. In addition, the activity of c-Src, a protein whose levels are enriched in lipid microdomains was required for ligand internalization by SREC-I [2, 33]. We therefore examined whether LPS could trigger transmembrane signaling through SREC-I/TLR4 by similar mechanisms. We first compared the intracellular localization of transfected SREC-I with that of a lipid microdomain marker, the GPI-anchored protein CD59; this protein is expressed in HeLa cells and internalized in a Cdc42 dependent manner into GEEC. For these experiments, FLAG-SREC-I was expressed in HeLa cells that were then incubated with LPS at 4°C for 30 minutes. LPS exposure led to SREC-I localization to a CD59-marked compartment on the plasma membrane (Fig 5A and 5B).

Next, we isolated detergent insoluble lipid microdomains/lipid raft fractions from LPS-treated or untreated Raw 264.7 cells. The lipid microdomainfractionsfrom cell lysates were prepared using *OptiPrep* layers and ultracentrifugation at ~200,000xg for 4 hours. Proteins isolated from such lipid microdomain fractions were then characterized by immunoblot assay (Fig 5C). SREC-I appeared to be absent from the detergent insoluble lipid microdomain fractions in controls, but in the presence of LPS was localized quantitatively to these fractions along with raft marker Flotillin, as well as, significantly, TLR4 (Fig 5C). However, there appeared to be little migration of CD14 (which is a GPI-anchored protein) into the microdomain fractionswith or without LPS (Fig 5C). As a further control we examined the behavior of endoplasmic reticulum-localized protein calnexin, which was observed to be absent from the lipid microdomain fractions with or without LPS (Fig 5C). These findings further suggested a role for sequestration in the lipid microdomains in LPS mediated SREC-I–TLR4 interactions.

### Role for entry into detergent insoluble plasma membrane lipid microdomainsin LPS-SREC-I-MAPK signaling

**Fig 6:**
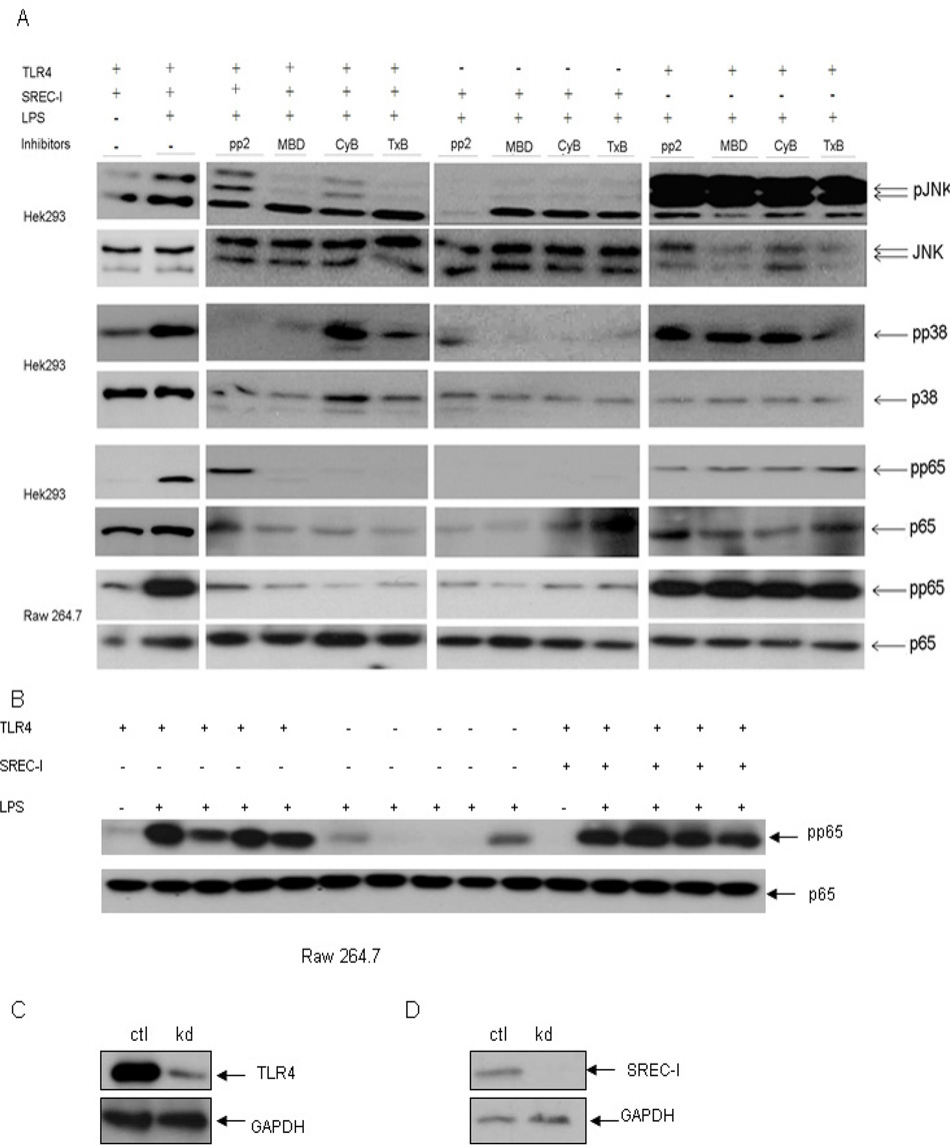
Intact lipid microdomains were essential for LPS-SREC-I-TLR4-induced MAPK signaling. **A,** HEK 293 cells expressing TLR4-MD2-CD14, SREC-I-TLR4-MD2-CD14 were treated with or without CD14 neutralizing peptide (inhibitor). Cells were then incubated with LPS (1 μg/ml) for 3 hrs. Cell lysates were run on SDS-PAGE and then immunoblotted with phospho-specific antibodies for JNK, p38, p65 and anti-JNK ab, p38 ab and anti-p65 ab. Drugs including PP2 (10 μM Srcinhibitor), MβCD (MBD, 10 mM, cholesterol sequestering agent), TxB (2 ng/ml, Clostridium Toxin B) were added to inhibit the functions of Src kinase, lipid microdomain formation and Rho GTPase activities as indicated. Raw 264.7 cells were transfected with siRNA for TLR4/SREC-I and then treated as described above. These experiments were repeated reproducibly 2 times. **B,** Raw 264.7 cells expressing TLR4, SREC-I or TLR4 only was incubated with LPS (1 μg/ml) for 3 hrs. Cell lysates were collected and then subjected to SDS-PAGE and Western Blotting. **C, D,** Raw 264.7 cells were transfected with indicated siRNA for 72 hours. Cell lysates were collected and equal amount of protein was subjected to SDS-PAGE and Western Blotting. Expression of SREC-I and TLR4 is shown in Raw 264.7 cells.

Next we examined the potential role of SREC-I localization to CD59-marked/lipid microdomainin TLR4 signaling through the JNK, p38 MAPK and NF-kB pathways using the HEK 293 system described above (Fig 6). We incubated cells with a number of agents known to disrupt lipid microdomains and formation of the GEEC compartment. These included cholesterol sequestering agent methyl β cyclodextrin (MβCD) and other inhibitors such as PP2 (Src kinase inhibitor), cytochalasin D (actin depolymerizing agent) and *Clostridium*. *Difficile* Toxin B (Rho GTPase inhibitor). These inhibitors each blocked internalization of antigen-bound SREC-I and subsequent antigen cross presentation in our prior studies [2]. In cells transfected with CD14 and TLR4, LPS induced efficient signaling through each pathway and these events were not markedly reduced by any of the inhibitors (Fig 6, 11^th^-14^th^ lanes). However LPS-induced activation of JNK and p38as well as NF-kB in cells with combined SREC-I/TLR4 expression (Fig 6, lanes 3–6) was blocked by many of the inhibitors (Fig 6). MβCD and Toxin B were particularly effective in this regard and blocked signaling through each pathway. Minimal signaling through the JNK, p38 or NF-kB pathways was observed in cells expressing SREC-I alone (Fig 6, lanes 7–10). This finding was predictable from the earlier experiments indicating a need for association with TLR4 in order for SREC-I to activate these pathways. This experiment suggested that TLR4 became co-localized with SREC-I after LPS exposure in new microdomains within the membrane and required enriched cholesterol, actin cytoskeleton function and small Rho GTPase activity for signaling pathways (Fig 6). When the experiments were repeated in Raw 264.7 cells, we again saw that inhibitors of lipid microdomain formation blocked NF-kB signaling in cells coordinately expressing TLR4 and SREC-I (Fig 6). As before, the inhibitors failed to disrupt NF-kB signaling in cells expressing TLR4 without SREC-I (Fig 6). These experiments, carried out in both HEK 293 and Raw 264.7 cells, indicated TLR4 signaling from either a lipid microdomain fraction or an internal endosomal compartment after SREC-I/TLR endocytosis via the GEEC pathway (Fig 6). We again confirmed the activation of NF-kB through phosphorylated p65 level in the presence of LPS in Raw 264.7 cells expressing TLR4 and SREC-I in the absence of inhibitors suggesting the role of lipid microdomain in activating LPS-SREC-I-TLR4 signaling (Fig 6B).

### Role of SREC-I in LPS-mediated inflammatory cytokine release

**Fig 7:**
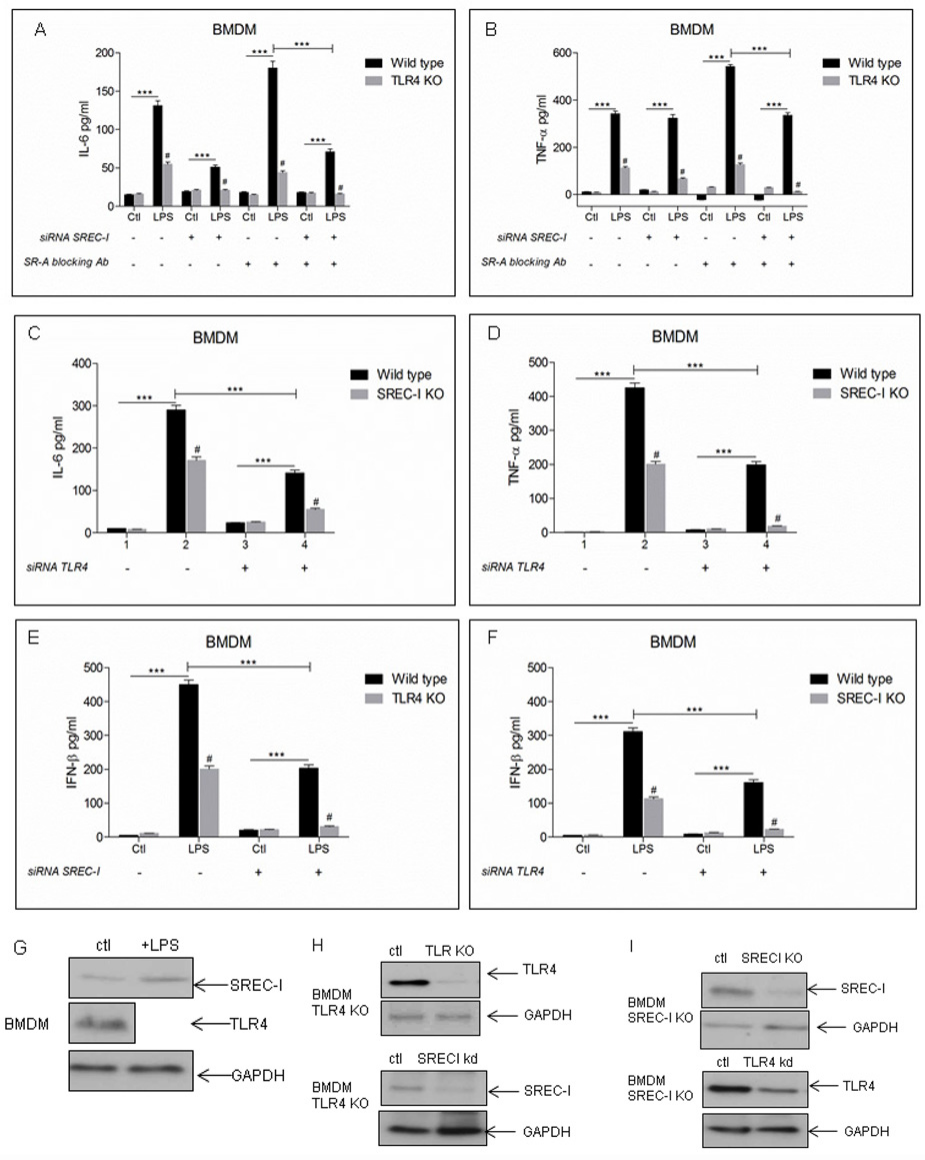
SREC-I inactivation reduced LPS-TLR4 mediated proinflammatory cytokine release in BMDM. **A,** TLR4 KO and SREC-I KD in macrophages led to reduced IL-6 release compared with WT cells. Primary macrophages from WT and TLR4 KO mice were transfected with siRNA-SREC-I or control hairpins for 72 hours and then incubated with LPS for 12 hours with or without SR-A blocking antibody. IL-6 release by cells was measured in the collected medium by ELISA. **B,** TLR4KO and SREC-I KD in BMDMs led to reduced TNF-α compared to WT cells. Primary macrophages from WT and TLR4 KO mice were transfected with siRNA-SREC-I or a control hairpin for 72 hours and then incubated with LPS for 12 hours with or without SR-A blocking antibody. TNF-α release was measured in medium by ELISA. **C,** SREC-I KO and TLR4KD in BMDM led to reduced IL-6 release compared with WT cells. Primary macrophages from WT and SREC-I KO mice were transfected with or without siRNA-TLR4 for 72 hours. IL-6 release into the medium was measured according to manufacturer's instructions. **F,** SREC-I KO and TLR4 KD BMDMs led to reduced release of TNF-α compared to WT cells. Primary macrophages from WT and SREC-I KO mice were transfected with or without siRNA-TLR4 for 72 hours. TNF-α release by was then assayed as above. **E,F**, TLR4 KO and SREC-I KO BMDMs led to reduced release of IFN-β compared to WT cells. Primary macrophages from WT and TLR4 KO (E), SREC-I KO were transfected or not with SREC-I siRNA (E) and TLR4 siRNA (F) for 72 hours. IFN-β release in media was assayed as above. **G, H, I,** Primary BMDM cells (WT, TLR4/SREC-I KO mice, TLR4/SREC-I KO + SREC-I siRNA/TLR4 siRNA) were trypsinized and cell lysates were collected. Equal amount of protein was subjected to SDS-PAGE and Western blotting. All experiments were performedreproducibly 3 times. Error bars in graph show S.D. between three replicate experiments. ***P<0.0001 when compared to the control, and #*P*<0.0001 when compared to the WT. Values were generated by ANOVA using the Bonferroni post-test.

As the above experiments indicated SREC-I mediated, TLR4 dependent signaling we next examined a role for this SR in LPS-induced cytokine production in bone marrow-derived mouse macrophages (BMDM). These cells express components of the LPS-TLR4 signaling apparatus, including CD14, MD2 and scavenger receptor A (SR-A) along with TLR4, TLR2 and SREC-I at physiological levels. We used both wild type bone marrow-derived macrophages as well as BMDM from TLR4 knockout and SREC-I knockout mice (Fig 7). BMDM were isolated and then separated from the total population of bone marrow derived cells using MAC-I and F4/80 macrophage specific antibodies (S2 Fig). We then measured the levels of interleukin-6 (IL-6, Fig 7A) and tumor necrosis factor α (TNF-α, Fig 7B) released by these macrophages. To examine a role for SREC-I expression in LPS-induced cytokine release, the BMDM were initially transfected with a SREC-I siRNA construct described previously [3]. As it was shown recently that LPS binding to scavenger receptor SR-A/CD204 could inhibit release of anti-inflammatory cytokines, we also assayed cytokine production either without or with (Fig 7A and 7B) blocking antibodies for this receptor [34]. LPS led to the induction of IL-6 (Fig 7A) and TNF-α (Fig 7C) in control cells and induction was markedly decreased by TLR4 KO. SREC-I knockdown (siRNA) also reduced the levels of LPS-mediated proinflammatory cytokine secretion in each case, while incubation with a control/scrambled RNA was ineffective (Fig 7A). Blocking antibodies for CD204/SR-A increased LPS-induced cytokine production in each case (IL-6 and TNF-α) (Fig 7A and 7B) [35]. In addition, IL-6 release was decreased in BMDM isolated from SREC-I knockout mice and further reduced by TLR4 siRNA (Fig 7C). We also assessed potential effects of SREC-I on LPS-induced TNF-α release in BMDM isolated from the SREC-I KO mice andsaw significant differences between WT and SREC-I KO mice (Fig 7D). TLR4 knockdown also reduced the levels of LPS-mediated IL-6 and TNF-α cytokine secretion in each case, while incubation with a control, scrambled RNA was ineffective (Fig 7C and 7D).

In addition to the inflammatory cytokine release, TLR4 has been shown to mediate induction of IFN expression in the presence of LPS. To determine a role for SREC-I we examined the release of IFN- β in BMDM taken from WT, SREC-I KO mice subjected to TLR4 knockdown with siRNA (Fig 7E). There was a sharp decrease in IFN-β release in the SREC-I KO/TLR4 knocked down BMDM (Fig 7F). Only low levels of IFN-β release were seen in cells taken from TLR4 KO-SREC-I knocked down BMDM indicating that SREC-I has a role in promoting LPS-TLR4 mediated IFN-β expression (Fig 7E).

**Fig 8:**
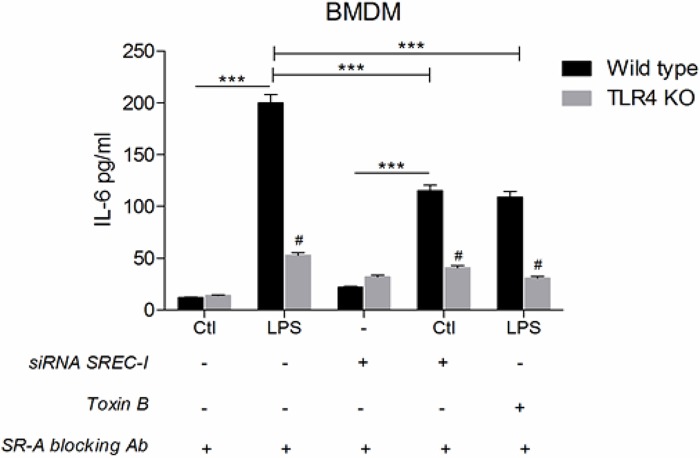
Rho GTPase activity was required for LPS-SREC-I-TLR4 induced proinflammatory cytokine release. BMDM cells from WT and TLR4 KO mice were transfected with siRNA SREC-I or control RNA. Cells were treated with or without Toxin B (2 ng/ml) and incubated withor without LPS. Experiments were performed in the presence of SR-A blocking antibodies. Cell media were collected, clarified by centrifugation and the IL-6 ELISA assay was performed. Experiments were repeated reproducibly 3 times. The height of the error bars represents the average of three independent measurements. The error bars represent one standard deviation from the mean. ***P<0.0001 when compared to the control, and #*P*<0.0001 when compared to the WT. Values were generated by ANOVA using the Bonferroni post-test.

Finally, we asked whether inhibiting the lipid microdomain formation/raft mediated internalization pathway for SREC-I localization could affect cytokine expression (Fig 8). Indeed, IL-6 release triggered by exposure to LPS was impaired when cells were treated with Toxin B, an inhibitor of the small GTPases (including Cdc42) required for the internalization pathway taken by SREC-I. The decrease in IL-6 expression was similar in magnitude to the decline in the cytokine observed when SREC-I was reduced in these cells by RNA interference (Figs 7 and 8). These results suggested that the nature of the lipid membrane microenvironment occupied by SREC-I after LPS treatment influenced TLR4 activity and downstream cytokine synthesis. To avoid the potential attenuation of cytokine release by SR-A, these experiments were performed in the presence of SR-A blocking antibody [35].

## Discussion

**Fig 9:**
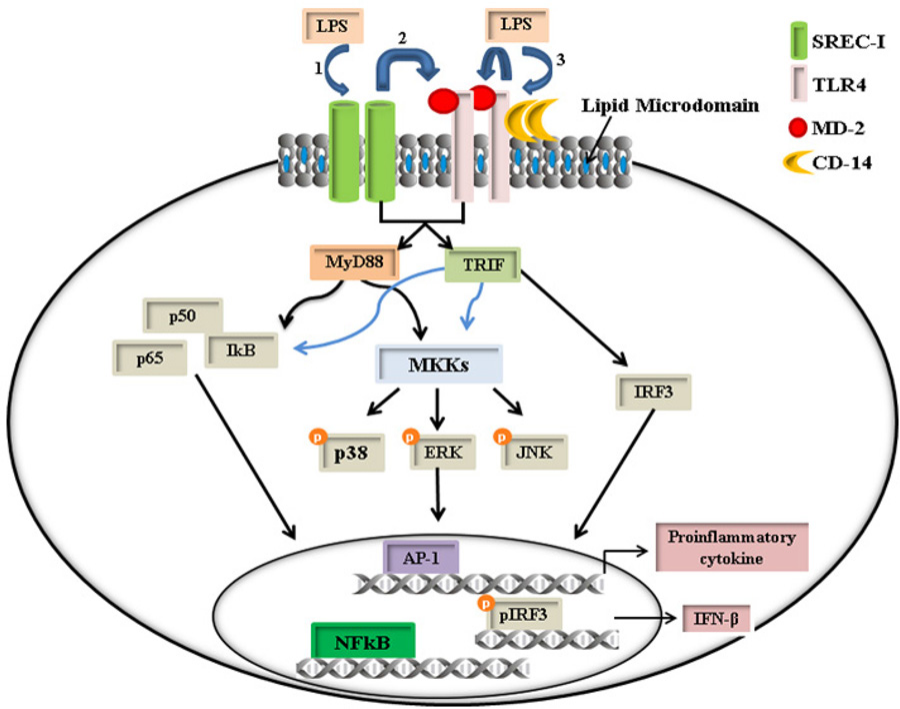
Schematic diagram of the proposed pathway of LPS-SREC-I-TLR4 mediated activation of proinflammatory cytokine expression. This cartoon depicts LPS binding to SREC-I (1) followed by LPS-SREC-I complex recruitment of TLR4 into discrete lipid microdomains (thick line) (2). Localization of LPS-SREC-I-TLR4 complexes to such lipid microdomains then led to activation of downstream proinflammatory cytokine release through adaptor proteins MyD88 and TRIF. LPS exposure could thus mediate activation of the NF-kB and MAPK pathways. In addition, LPS has been shown to be recognized by CD14, the primary responder to the endotoxin, which then bound to TLR4 and triggered proinflammatory signaling through NF-kB and MAPK (3). We have additionally shown that LPS-SREC-I-TLR4 signaling led to activation of IRF3. Engagement of these signaling pathways could then lead to activation of transcription factors NF-kB, AP-1 and IRF3 that have been shown to function combinatorially in cytokine gene transcription.

Our experiments therefore have shown SREC-I to be a receptor capable of responding to LPS and interacting with TLR4 to trigger inflammatory signaling leading to enhancement of TNF-α, IFN-β and IL-6 expression (Fig 9). The mechanisms involved in the enhancement of cytokine secretion by SREC-I may involve sustained and stronger activation of the NF-kB and MAP kinase pathways that are known to function upstream of cytokine expression (Figs 2 and 3). Previous studies had suggested that SREC-I could interact with an extracellular protein-Tamm-Horsfall protein that may mediate TLR4 dependent inflammatory responses in dendritic cells [36]. Although SREC-I was shown to cooperate with TLR2 in recognition of hepatitis C virus N3 proteins in myeloid cells [37], we did not observe TLR2 involvement in LPS mediated TLR4 signaling by SREC-I (S3 Fig). We hypothesize that the mechanisms utilized for the mediation of TLR4 downstream signaling by SREC-I interaction appeared to involve LPS binding and recruitment of TLR4 into the GEEC pathway of internalization [32, 38] by ligand-bound SREC-I, a process initiated by migration of the SREC-I into cholesterol rich lipid microdomains (Fig 6). Cholesterol-rich microdomains were shown to contain abundant levels of signaling molecules and indeed ligand binding to SREC-I promoted functional association with non-receptor tyrosine kinase Src and the small GTPase, Cdc42; these processes were shown to induce internalization in a dynamin-independent manner [2, 4, 33, 39]. Our experiments suggested that SREC-I might be able to function in some circumstances as a primary LPS receptor in a manner reminiscent of CD14, although the latter receptor has not been shown to assist in internalization of TLR4 into GEEC. Instead, CD14 appeared to promote TLR4 endocytosis via a dynamin-dependent pathway that was independent of Src and Cdc42 but dependent on another non-receptor tyrosine kinase, Syk [13, 40]. Our experiments therefore indicated that, as well as being involved in antigen cross presentation and adaptive immunity, SREC-I could stimulate innate immune processes by co-opting the activity of TLR4. So far the known regulators of TLR4 endocytosis include dynamin, clathrin and all other associated proteins. We cannot rule out however the possibility of other specialized means of microbial recognition by cells that are coupled to TLR4 signaling. In this regard, SREC-I mediated LPS-TLR4 binding and signaling involving insoluble lipid microdomains could be significant (Fig 5). SREC-I might be activated in this way by LPS (Figs 5 and 6), or any potentially by other microbial products, which are also involved in recognition microorganisms (A. Murshid, unpublished data).

CD14 was originally identified as a key factor in MyD88-dependent signal transduction at very low concentrations of LPS [41, 42]. Our findings suggested that SREC-I could supplement CD14 as a factor in MyD88-dependent signal transduction and it could also facilitate TRIF signaling at somewhat higher concentration of LPS both from plasma membrane and endosomes respectively in HEK 293 cells expressing SREC-I and TLR4. Earlier it was shown that the scavenger receptor CD36 and the mannose receptor could serve as alternatives to CD14 for TLR2 induced signaling [43]. In an analogous manner, SREC-I might participate in LPS-TLR4 signaling in addition to CD14 activity (Figs 2 and 7).

Structurally distinct SR family members, SREC-I and SR-A/CD204 have been ascribed number of properties in common in addition to binding LPS. These properties include the capacity to associate with modified proteins and to bind a number of heat shock proteins [44–47]. Both receptors were increased in expression in activated macrophages [48] (Calderwood SK, unpublished data). However, while SREC-I promoted antigen cross presentation, activation of CTL (cytotoxic T cell) and induction of innate immunity [2, 49] (Figs 3 and 7), the effects of SR-A/CD204 were anti-inflammatory and this receptor could inhibit a number of mechanisms in innate and adaptive immunity [50–52]. Some of these contrasting effects could involve the influence of SR expression on TLR4 activity; whereas SREC-I enhanced LPS-TLR4 signaling, at least partially through sequestration of the TLR in lipid microdomains (Figs 5 and 6), SR-A/CD204 was shown to inhibit the ubiquitinylation of the adaptor protein TRAF6, thus reducing levels of NF-kB signaling [53]

One unexplained finding in our studies was that Hsp90, although binding avidly to SREC-I and stimulating TLR4 association caused relatively low levels of inflammatory signaling. HSPs have been proposed as potential endogenous danger signals [54, 55]. One finding that could account for the paucity of Hsp90 induced inflammatory signals found here is that HSPs could interact with SR-A/CD204, a receptor that has been shown to inhibit TLR4 signaling [34]. However, SR-A also dampened LPS induce TLR4 signaling [34]. The differences between LPS and Hsp90 in terms of signaling through SREC-I were thus not certain. However, the extracellular component of SREC-I is a large multidomain structure and has been shown to contain numerous distinct potential sites for different ligands [4]. Future studies would address this question.

In conclusion therefore, we hypothesize that the Class F scavenger receptor SREC-I became associated with TLR4 within lipid microdomainsin Raw 264.7 macrophagesexposed to LPS, triggered inflammatory signaling and promoted sustained cytokine release (Fig 9). Earlier it has been shown that this receptor, expressed in both macrophages and DC, interacts with a range of ligands in addition to LPS and could thus participate in innate and adaptive responses to a range of endogenous or pathogenic immune challenges.

## Supporting Information

S1 Fig SREC-I expression led to enhanced ERK 2/1 activities in cells expressing TLR4 in the presence of LPS. **A,** SREC-I could increase LPS-TLR4 activation of MAPK. HEK 293 cells expressing TLR4-MD2-CD14, TLR4-MD2-CD14-SREC-I were incubated with LPS (1 μg/ml) for 5 hours (CD14 neutralizing peptide added to SREC-I incubation). Cell lysates were collected and levels of phosphorylated ERK1/2 MAPK assayed.
(TIF)

S2 Fig BMDM were isolated from WT and KO mice. **A,** Bone marrow cells were isolated and differentiated to macrophages. Cells were then stained with anti F4/80 antibody.
(TIF)

S3 Fig SREC-I supported LPS-TLR4 mediated NF-kB (phospho-p65). **A,** HEK 293 cells expressing SREC-I and TLR4, TLR2 +SREC-I or TLR4 only were transfected with NF-kB-SEAP and incubated with LPS (1 μg/ml) or Pam3CSK4 (10 μg/ml) for 5 hours. NF-kB activity was measured as instructed by NF-kB-SEAporter assay kit.
(TIF)
