# Supplementary figures and images for: Scavenger Receptor SREC-I Mediated Entry of TLR4 into Lipid Microdomains and Triggered Inflammatory Cytokine Release in RAW 264.7 Cells upon LPS Activation
Source: PLoS One. 2015 Apr 2;10(4):e0122529. doi: 10.1371/journal.pone.0122529 (PMC4383338; doi:10.1371/journal.pone.0122529)

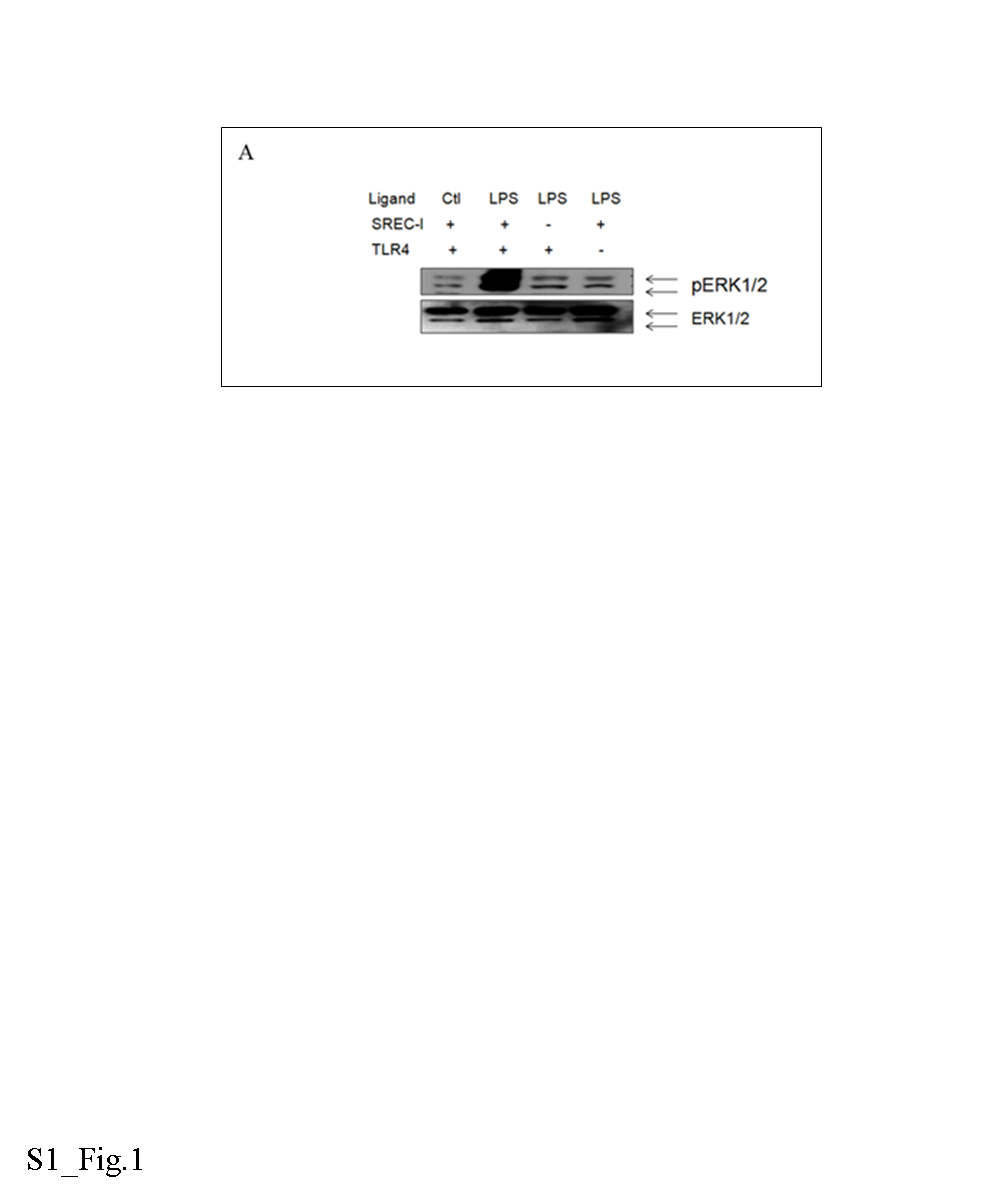

Supplement: S1 Fig — A, SREC-I could increase LPS-TLR4 activation of MAPK. HEK 293 cells expressing TLR4-MD2-CD14, TLR4-MD2-CD14-SREC-I were incubated with LPS (1 μg/ml) for 5 hours (CD14 neutralizing peptide added to SREC-I incubation). Cell lysates were collected and levels of phosphorylated ERK1/2 MAPK assayed. (TIF) [file pone.0122529.s001.tif]

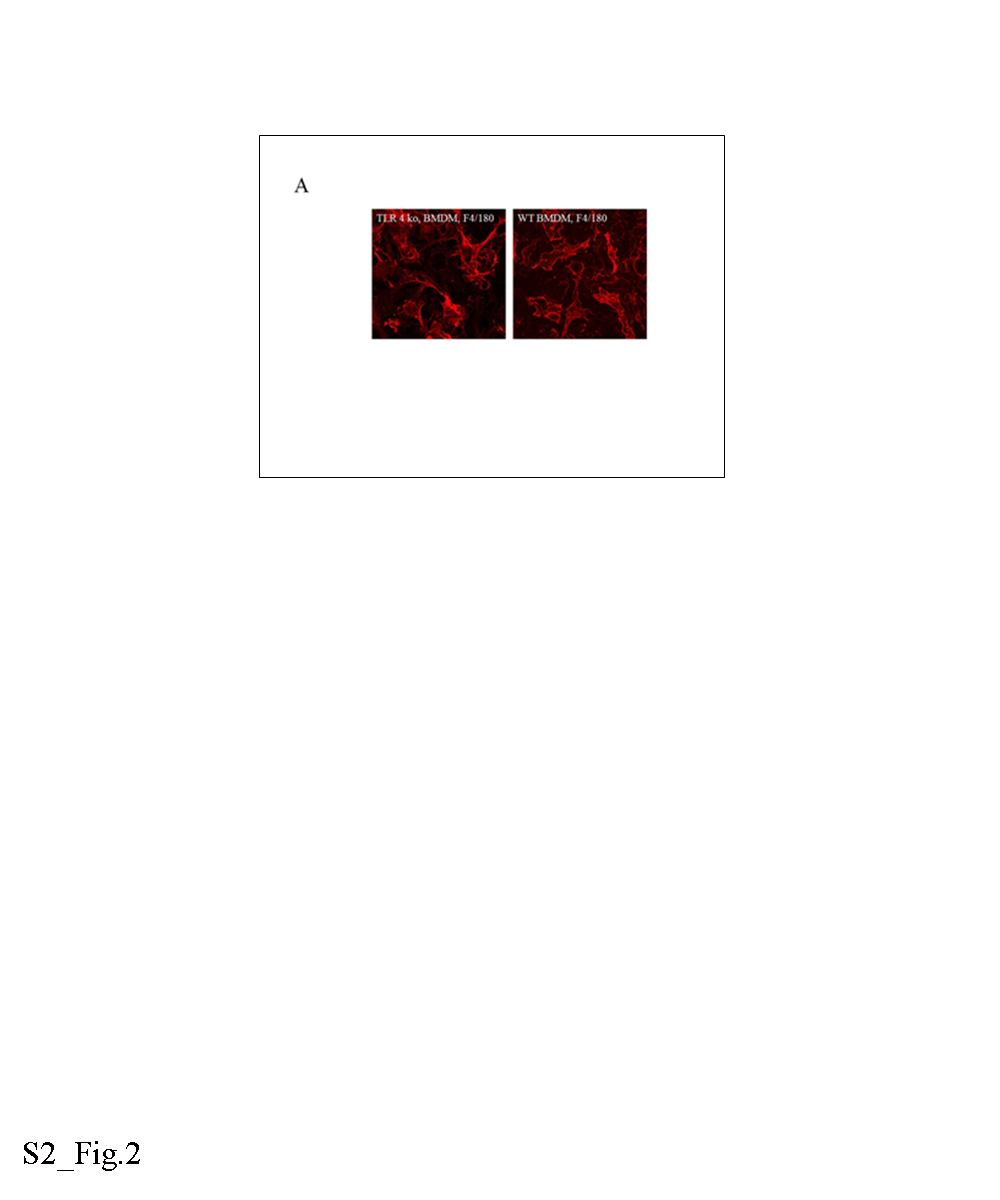

Supplement: S2 Fig — A, Bone marrow cells were isolated and differentiated to macrophages. Cells were then stained with anti F4/80 antibody. (TIF) [file pone.0122529.s002.tif]

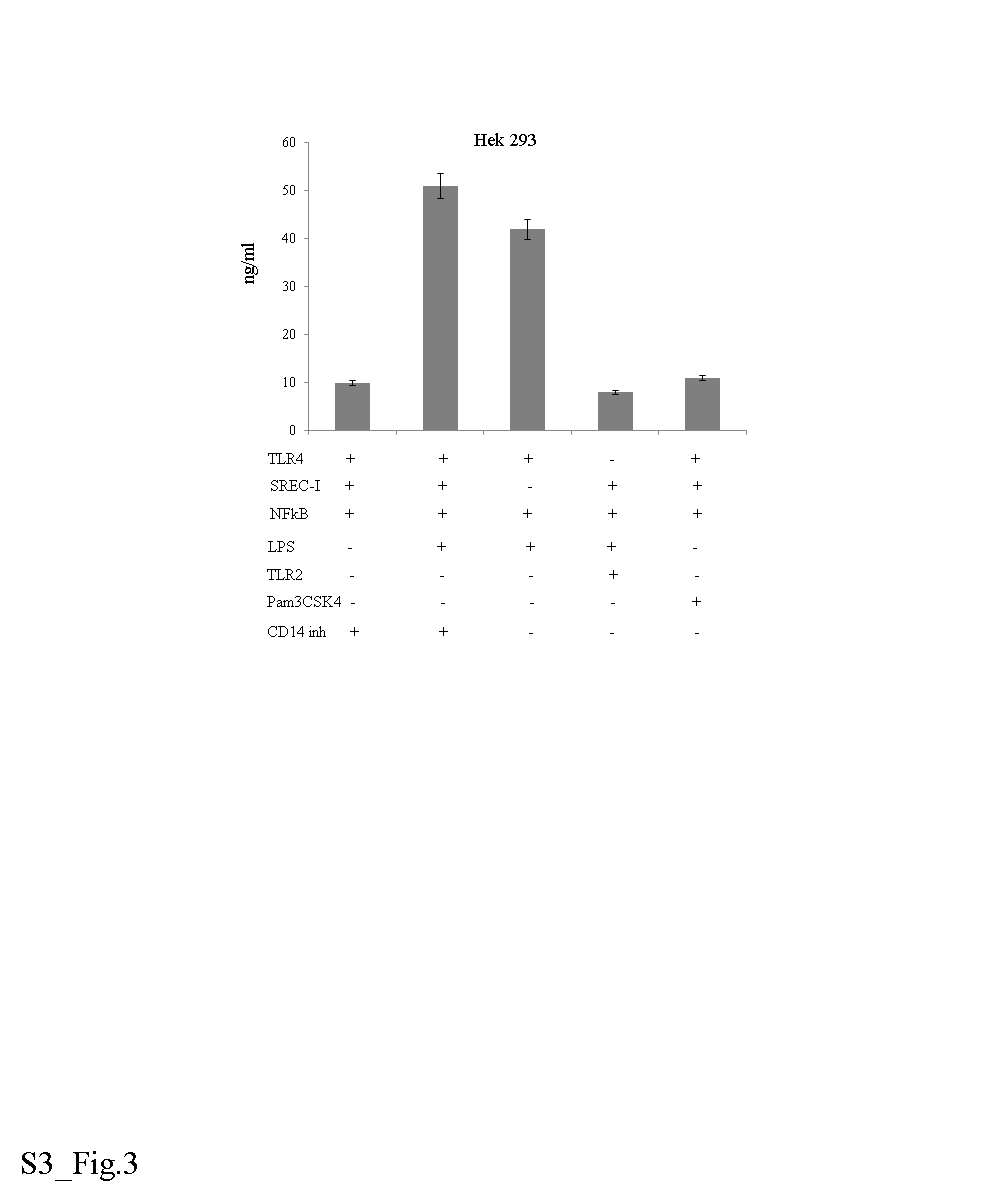

Supplement: S3 Fig — A, HEK 293 cells expressing SREC-I and TLR4, TLR2 +SREC-I or TLR4 only were transfected with NF-kB-SEAP and incubated with LPS (1 μg/ml) or Pam3CSK4 (10 μg/ml) for 5 hours. NF-kB activity was measured as instructed by NF-kB-SEAporter assay kit. (TIF) [file pone.0122529.s003.tif]
